# Phosphorylation of YBX1 in the Kidneys is Altered in Legumain Knockout-Mice

**DOI:** 10.1021/acs.jproteome.6c00105

**Published:** 2026-05-06

**Authors:** Tilen Sever, Tea Sinožić, Matej Kolarič, Boris Turk, Marko Fonović

**Affiliations:** † 61790 Jožef Stefan Institute, Department of Biochemistry, Molecular and Structural Biology, Jamova cesta 39, Ljubljana SI-1000, Slovenia; ‡ International Postgraduate School Jožef Stefan, Jamova cesta 39, Ljubljana SI-1000, Slovenia; § University of Ljubljana, Faculty of Medicine, Vrazov trg 2, Ljubljana SI-1000, Slovenia; ∥ University of Ljubljana, Faculty of Chemistry and Chemical Technology, Večna pot 113, Ljubljana SI-1000, Slovenia

**Keywords:** legumain, YBX1, phosphorylation, knockout mice

## Abstract

Protein phosphorylation is a common post-translational modification that plays a crucial role in cellular signal transduction. Disruptions in this process can lead to phenotypic deviations in healthy organisms. Legumain is a cysteine proteinase present in plants and animals. Legumain is involved in the regulation of kidney and hematopoietic homeostasis, as well as immune response. Its dysregulation is associated with various types of cancers and neurodegenerative diseases. Legumain knockout mice generally exhibit a normal phenotype, except for altered kidney function, hemophagocytic syndrome, and extramedullary hematopoiesis. In this study, we analyzed the changes in protein phosphorylation in legumain knockout mice compared to their wild-type counterparts to elucidate how legumain deficiency affects protein phosphorylation and related cell signaling. Phosphopeptides from the kidney and liver samples were enriched and analyzed using mass spectrometry and validated with Western blot and immunohistochemistry. Several phosphorylation sites on the RNA- and DNA-binding protein Y-box binding protein 1 were identified. A site on the serine 100 residue was found to activate the NF-κB pathway in legumain knockout mice, resulting in an enhanced inflammatory response. This was supported by the increased expression of several NF-κB genes. Overall, this study provides valuable insights into the role of legumain and its impact on various cellular processes.

## Graphical abstract

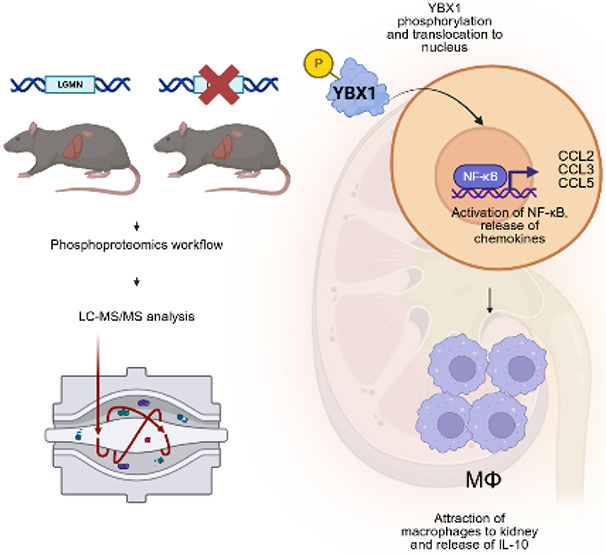

## Introduction

Legumain, also known as asparagine endopeptidase (AEP), is a cysteine proteinase (family C13, CD 3.4.22.34)^1^ found in plants and animals. In mammals, it is expressed in a wide array of tissues but is most abundantly expressed in the kidneys, testes, placenta, and spleen.^2^ The protease has a high cleavage specificity at the C-terminal of asparagine and aspartate residues, with an optimum pH of approximately 5.8.^3,4^ In immune cells of hematopoietic origin,^5^ legumain is involved in the regulation of hematopoietic homeostasis and immune responses. This is regulated by the presentation of antigens with MHC II,^6^ the processing and activation of Toll-Like Receptors 7 and 9,^7^ monocyte differentiation and maturation^8,9^ and the stability of the erythrocyte membrane.^10^ Legumain affects bone remodeling by inhibiting osteoclast formation^11^ and differentiation.^12^ It is also commonly found to be dysregulated in breast,^13^ colo-rectal,^14^ ovarian,^15^ and prostate cancer.^16^ Despite the recent identification of legumain substrates in murine spleen,^17^ it remains unclear how legumain affects these processes.

Mice without legumain expression exhibited a mild phenotype with no behavioral aberrations, normal fertility, and viability.^10^ However, several changes, such as altered morphology and function, have been reported in the kidneys of knockout mice. For instance, the accumulation of endosomal proteins in proximal tubular cells results in enlargement of endosomes and hyperplasia of these cells. Interstitial fibrosis and glomerular cyst formation were observed. Changes in kidney function include reduced glomerular filtration rate, elevated plasma creatinine levels, and proteinuria.^6,18^ In relation to hemopoiesis, knockout mice exhibit several characteristics associated with the hemophagocytic syndrome, including histiocytes containing red blood cells, a reduction in hematocrit values as they age, and an enlarged spleen with active extramedullary hematopoiesis. Knockout mice showed greater resistance to ischemia-induced brain injury^19^ and slower progression of Alzheimer’s disease, where phosphorylation of the microtubule-associated protein tau was observed.^20,21^ Legumain deficiency also represses depression- and anxiety-like behaviors in mice.^22^ Although the livers of the knockout animals appeared normal histologically, they had larger, darker organs compared to the wild type.^10^ This study is the first to show how protease deficiencies, such as legumain deficiency, affect cell signaling through protein phosphorylation.

Protein phosphorylation is a common post-translational modification that fundamentally alters the biochemical properties of target proteins by converting them from hydrophobic to hydrophilic polar molecules. This change in polarity enables conformational modifications that can either activate or deactivate protein functions, influence protein–protein interactions, or initiate subcellular relocalization.^23^ The phospho-rylation status of proteins is dynamically regulated by two opposing enzyme families: protein kinases, which catalyze phosphorylation, and protein phosphatases, which remove phosphate groups.^24^ Protein phosphorylation plays a key role in various biological processes, including signal transduction, metabolism, and cell cycle regulation. Irregularities in protein phosphorylation are observed in numerous pathological conditions, including cancer and neurodegenerative diseases.^23^

Y-box binding protein 1 (YBX1) is a highly versatile and evolutionarily conserved protein that acts as a key regulator of cellular gene expression. As part of the cold shock domain (CSD) protein family, YBX1 is crucial for virtually all cellular functions, ranging from basic RNA processing to complex disease pathogenesis, making it one of the most intensively studied proteins in molecular biology.^25^ YBX1 undergoes extensive post-translational modifications, with phosphorylation being the most prevalent and functionally significant. Phosphorylation occurs at multiple sites in the protein and is a critical regulatory mechanism that influences YBX1’s subcellular localization, protein interactions, and diverse cellular functions.^26,27^

Altered protein phosphorylation has been linked to some of the changes observed in legumain knockout mice,^28–35^ along with increased epidermal growth factor receptor (EGFR) in the kidneys^6^ and signal transducer and activator of transcription 3 (STAT3) signaling.^36^ Additionally, phosphorylation of the pyruvate dehydrogenase (PDH) domain of podocin was found to influence its dimerization and, therefore, podocyte filtration function in murine kidneys.^29^ In renal interstitial fibrosis models, the phosphorylation of p38 mitogen-activated protein kinase (MAPK) is increased by the nuclear receptor Nr4a1, which exacerbates renal fibrosis.^37^ The JAK/STAT signaling pathway, which depends on phosphorylation for activation, has been identified as a key driving factor in myelofibrosis and is directly linked to extramedullary hematopoiesis.^38^ Increased STAT1 phosphorylation has been observed in patients with hemophagocytic lymphohistiocytosis.^39^ Hepatomegaly has been observed after activation of Nuclear factor (erythroid-derived 2)-like 2, a transcription factor that controls antioxidant expression and is activated by AKT kinase.^40^

In this study, we analyzed the changes in protein phosphorylation between legumain knockout mice and their wild-type counterparts. Since legumain is predominantly expressed in the kidneys and liver,^41^ our research focused on these two organs to understand the impact of legumain deficiency on cellular signaling. Protein phosphorylation is a likely mediator between legumain and the changes observed in legumain knockout mice. This study aimed to elucidate the phosphorylation sites involved in activating the pathways that amplify the inflammatory responses in the kidneys and livers of mice. The results provide valuable insights into the role of legumain in the organism and its impact on cellular processes.

## Materials and Methods

### Phosphopeptide Enrichment and Identification by Mass Spectrometry

Kidneys and liver were harvested from WT and *Lgmn* ^*–/–*^ (n= 3 for each genotype) mice with C57/BL6 background and immediately frozen in liquid nitrogen. The animals were maintained according to national regulations approved by the Veterinary Administration of the Republic of Slovenia (VARS) and the government Ethical Committee (permit number U34401–40/2020/3). Mice (five animals per cage) were housed in standard cages (Techniplast 1264C Eurostandard Type II) with corncob bedding (Rehofix MK 2000) and water and food (Altromin 1324) were provided ad libitum. Tissue lysates were prepared using a lysis buffer (50 mM Tris-HCl, 150 mM NaCl, 1m M EDTA, 0.5% Nonidet P-40 (v/v), 0.5% potassium deoxycholate (v/v), 0.1% SDS (v/v), and pH 7.4), 20 μL of buffer per 1 mg of tissue, with added phosphatase inhibitor cocktail (G-Biosciences). The tissues were manually homogenized with a Potter-Elvehjem PTFE homogenizer on ice, and the homogenates were shaken on ice for an additional 30 min. The samples were then centrifuged at 12000 × g for 30 min at 4 °C, and the supernatants were collected and used immediately. Total protein concentration was determined using the Bradford assay and 2 mg of total proteins were used for in-solution digestion. Urea was added to the samples to a final concentration of 6 M. For reduction, dithiothreitol (DTT) was added (10 mM final concentration), and samples were incubated at room temperature (22–25 °C) for 1 h. The reduced proteins were alkylated with iodoacetamide (32 mM) in the dark at room temperature for 1 h. Subsequently, the reaction was quenched by the addition of DTT (38 mM) and incubated for 1 h at room temperature. Proteins were digested with 1:100 (w/w) sequencing grade Lys-C/Trypsin mix (Promega) for 3 h at 37 °C, after which samples were diluted to urea concentration of 1 M with 50 mM Tris-HCl, 0.1% SDS (v/v), pH 7.4 and digestion continued overnight at 37 °C. After digestion, the reaction was quenched by adding trifluoroacetic acid (TFA, 0.5%) and samples were centrifuged at 12000 × g for 15 min to remove debris. Supernatants were then concentrated on C18 reverse phase columns (8B–S001-DAK, Phenomenex). Columns were equilibrated with three volumes of methanol, 0.1% formic acid in acetonitrile, and 0.1% formic acid in water. After loading, samples were washed with three volumes of 0.1% formic acid and eluted with 400 μL of 60% acetonitrile with 0.1% formic acid and concentrated with SpeedVac. Concentrated samples were reconstituted in 0.1% TFA and 50% acetonitrile to approximate protein concentration of 4 μg/μL. Subsequently, phosphopeptides were enriched using PHOS-Select Iron Affinity Gel (Sigma-Aldrich). A suspension of PHOS-Select beads (40 μL) was placed in SigmaPrep spin columns (SC1000–1KT, Sigma-Aldrich) and rinsed three times with a solution of 0.1% TFA, 50% acetonitrile. The samples and beads were then transferred to SigmaPrep columns and mixed for 1 h at room temperature. After incubation, flow-through was collected as the unbound fraction, and the gel was washed twice with 200 μL of 1% TFA, 35% acetonitrile. The eluate from this step was pooled with the unbound fraction and concentrated using a SpeedVac for further enrichment of phosphopeptides with Titansphere Phos-TiO_2_ beads (5010–21315, GL Sciences). The iron affinity gel was rinsed with 250 μL of LC-MS grade water, and phosphopeptides were eluted using 150 mM NH_4_OH and 25% acetonitrile while shaking for 5 min. The eluted phosphopeptides were concentrated in a SpeedVac and transferred to a C18 StageTips (20 discs, 3M Empore). The stage tips were equilibrated as previously described with C18 columns. Elution from the StageTips was performed sequentially with 100 μL of elution buffer containing 0, 2, 5, 8, 10, and 40% acetonitrile in 0.1% formic acid at 1400 × g for 5 min. The concentrated unbound fraction from the iron affinity gel was reconstituted in a solution of 1 M glycolic acid, 80% acetonitrile, and 5% TFA to an approximate concentration of 4 μg/μL, while TiO_2_ beads were prewashed three times with 80% acetonitrile and 0.4% TFA. The beads were added to the samples at a ratio of 1:8, w/w, and mixed for 1 h at room temperature. After incubation, the samples were spun down, and the beads were transferred to C18 StageTips, which were prepared with 20 C18 discs in 200 μL tips and equilibrated as previously described with C18 columns. The samples were washed with 100 μL of 0.5% TFA, 30% acetonitrile, 0.4% TFA, and 80% acetonitrile, respectively. Sequential elution was performed with 0, 2, 5, 8, 10, and 40% acetonitrile in 15% ammonia. All eluted samples were concentrated in a SpeedVac and reconstituted in 0.1% formic acid in water for LC-MS analysis.

Samples were separated online using an EASY-nano LC II HPLC system equipped with a C18 trapping column (Proxeon EASY Columns, Thermo Fisher Scientific) and a C18 analytical column (PicoFrit AQUASIL, New Objective). Peptide elution was achieved with a 90-min linear gradient (buffer A: 0.1% formic acid in water, buffer B: 0.1% formic acid in acetonitrile) from 5% to 50% buffer B at a flow rate of 300 nL/min. Proteins were identified using an Orbitrap LTQ Velos mass spectrometer (Thermo Fisher Scientific), set to positive ion mode. Full mass spectra were recorded at 30000 resolution between 300 and 2000 *m*/*z*. The nine most intense ions with a charge greater than 1 were chosen for MS/MS scans using higher-energy collisional dissociation (HCD) fragmentation. Dynamic exclusion was activated, allowing for a single repeat with a duration of 30 s, and an exclusion duration of 30 s. The MS/MS spectra were recorded with a resolution of 7500 using Thermo Xcalibur ver. 2.2 SP1.48 (released on August 11, 2012).

A control experiment was conducted to confirm that the observed changes were solely due to variations in protein phosphorylation and not due to differences in overall protein abundance. The experiment was performed as described in Sever et al.,^42^ but omitted the use of *N*-hydroxysuccinimide ester of trideutero-acetate labeling.

### Western Blotting

Murine kidney and liver samples were homogenized as described in the previous paragraph. After determining and equalizing total protein concentration, the samples were heated at 95 °C for 5 min in SDS-PAGE loading buffer. Total proteins (20 μg) were separated using 10% or 12% SDS-PAGE and then transferred onto a nitrocellulose membrane using the Trans-Blot Turbo (Bio-Rad) for 10 min at 2.5 A, 25 V, according to manufacturer’s instructions. The membranes were briefly washed in TBS buffer with 1% Tween-20 (TBS-T) and blocked with 5% skim milk in TBS-T for 1 h at room temperature. Primary antibodies were applied to the membrane and incubated overnight at 4 °C. After incubation, the membranes were washed four times with TBS-T and incubated with HRP-conjugated secondary antibodies for 1 h at room temperature, and then washed again four times with TBS-T before being imaged with enhanced chemiluminescent (ECL) Western Blotting Detection Reagent (GE Healthcare) and the G: BOX Chemi XRQ gel dock system (Syngene). The blots were normalized on loading control and then quantified with the help of ImageJ software (ver. 1.49). Blots for phosphorylated proteins were first normalized to the loading control and then to the normalized whole protein. The antibodies used included: anti-GAPDH (Cell Signaling Technology, 97166S), anti-IkBα (Cell Signaling Technology, 9241), anti-IL-10 (Abcam, ab9722), anti-YBX-1 (Abcam, ab12148), anti-pSer 100 YBX-1 (Abcam, ab138654), and goat antirabbit secondary antibodies with horseradish peroxidase (Jackson ImmunoResearch, 111–035–045).

### Mammalian Expression

To express legumain in mammalian cells, the legumain cDNA was inserted into the pLVX-IRES-Puro lentiviral expression vector (Clontech). Lentiviruses were produced using the Lenti-X lentiviral expression system (Invitrogen), according to manufacturer’s instructions. Briefly, the expression vector was transfected into Lenti-X 293T cells along with the Lenti HTX Packaging Mix to create infectious virions. These recombinant virions were then used to infect a mammalian cell line (Human leukemia 60, HL-60) and generate legumain-expressing cells, as described by the manufacturer (Invitrogen). The primers used for constructing the expression vector were: 5LegEcoR1 (5′-TTAATTGAATTCATGGTTTGGAAAGTA-3′) and 3LegXba1 (5′-TTAATTTCTAGATCAGTAGTGACCAAG-3′).

HL-60 cells lacking legumain expression were purchased from ATCC (98070106). The cells were cultured in RPMI 1640 medium with 20% fetal bovine serum (FBS), 1% penicillin/streptavidin (all from Sigma-Aldrich), and 1% GlutaMAX (Invitrogen). The cells were cultured in a humidified incubator at 37 °C and 5% CO_2_.

Cell lysates were prepared in lysis buffer (50 mM Tris-HCl, 150 mM NaCl, 1 mM EDTA, 0.5% Nonidet P-40, 0.5% potassium deoxycholate, 0.1% SDS, pH 7.4), to which phosphatase inhibitor cocktail (G-Biosciences) and protease inhibitor cocktail (Sigma) were added. The cells were manually homogenized on ice using a syringe with a 20G needle, and the homogenates were shaken on ice for a further 30 min. Samples were then centrifuged at 12000 × g for 30 min at 4 °C. The supernatants were collected, and the total protein concentration was determined using the Bradford assay.

### RT-qPCR

Total RNA was extracted from kidney and liver samples and isolated using the QIAGEN RNeasy Mini Kit (Qiagen) as per manufacturer’s instructions. The isolated RNA concentration was determined by using a NanoDrop One/One spectrophotometer (Thermo Scientific). In the reverse transcription reaction, 1 mg of total RNA was used with LunaScript RT Supermix (New England Biolabs). For the RT-qPCR reaction, 10 ng of cDNA per well was used with KAPA SYBR FAST (Merck) master mix. The primers were used at a final concentration of 100 nM. The RT-qPCR was conducted with a Stratagene MX300P (Agilent) with the following settings: an initial (i) denaturation at 95 °C for 3 min, (ii) 40 cycles of denaturation at 95 °C for 3 s, and primer annealing and extension (60 °C at 20 s). Data were collected at the end of each cycle, and the melting curve was assessed after thermal cycling was complete. A set of housekeeping genes was evaluated for expression stability across all samples using the GNorm method^43^ with GUSB identified as the most stable reference gene. The results were analyzed in R using the pcr package (ver. 1.2.2)^44^ and the double delta Ct method.^45^ Primers were purchased from Integrated DNA Technologies and are listed in Supplement Table S1.

### Phosphoproteomic Data Analysis

The data files generated by the LTQ Orbitrap Velos were processed by using MaxQuant software (version 1.6.17.0, Max-Planck Institute for Biochemistry) with a built-in Andromeda search engine. The files were compared against the reviewed Swiss-Prot *Mus musculus* database with 16996 entries as of August 2019. All parameters were kept at their default values, except for the addition of STY phosphorylation as a variable modification and the activation of Label-Free Quantification. Results from MaxQuant were further analyzed using Perseus software (ver. 1.6.14). Before processing, potential contaminants, reverse hits, and phosphosites with a localization probability below 0.75 were excluded. Only phosphosites identified in at least two out of three technical and three biological replicates each were retained for the Student’s *t*-test. A permutation-based FDR was used for truncation, with the FDR set to 1%.

### Immunofluorescence Staining

Mouse kidneys were frozen in liquid nitrogen immediately after dissection and stored at −80 °C. The organs were cut into 5 μm sections by using a cryostat (SLEE Medical) and mounted on glass slides. Tissue samples were fixed using 4% cold paraformaldehyde and 100% cold acetone. For immunofluorescence labeling, anti-F4/80 primary antibodies (diluted 1:100, Abcam, ab6640) were used, followed by secondary antibodies Alexa Fluor 555 antirat IgG (diluted 1:200, Invitrogen, A48270). Nuclear counterstaining was performed using an antifade mounting solution containing 4′,6-diamidino-2-phenylindole (DAPI) (Thermo Fisher, P36931), and the glass slides were covered with coverslips. A fluorescence microscope (Olympus IX 81) was used to capture images of the tissue samples, and the Olympus cellSens Dimension (version 3.2) imaging software was used for image analysis. Five regions per sample were chosen, and the percentage of the stained areas was quantified after adjusting the color threshold and normalizing the signal to the DAPI signal using ImageJ software (ver. 1.49).

## Results

Legumain knockout mice exhibited a mild phenotype but with altered kidney function, heightened immune response, and extramedullary hematopoiesis. We investigated changes in protein phosphorylation between wild-type and legumain-knockout mice to determine the precise mechanisms affecting the phosphorylation status of key signaling proteins involved in these physiological alterations.

### Phosphoproteomic Profile Analysis

We identified 5082 phosphosites in the kidneys, with 3089 of these having peptide identification confidence of at least 99% and site localization confidence exceeding 75% (as recommended).^46^ Altogether, we detected 3183 phosphosites located on 1681 proteins. The majority of phosphorylated peptides (3827, 91.5%) were phosphorylated at a single site, 332 (7.9%) were phosphorylated at two sites, and 24 were phosphorylated at least on 3 residues. The majority of the sites (82.0%) were located on serine residues, whereas 16.7% and 1.3% were located on threonine and tyrosine residues, respectively. Among the phosphosites identified, 31 were differentially regulated between the knockout and control groups. Of these, only YBX1 and Srrm2 were multiphosphorylated (Figure S1A, Supplemental Figure S1A, Supplemental Table S2).

In the liver, 2912 phosphosites were identified, of which 1842 exceeded 75% localization confidence (class I sites). These phosphosites were found on 2268 phosphopeptides, which were linked to 1154 proteins. Similar to the findings in the kidneys, the majority of phosphopeptides were phosphorylated at a single site (1954, 85.9%), whereas 275 (12.0%) were phosphorylated at two sites, and 47 (2.0%) were phosphorylated on at least three residues. Among the identified phosphorylation sites, 82.9% (2413) of them were phosphorylated on serine residues, while 16.1% (469) and 1.0% (30) were phosphorylated on threonine and tyrosine residues, respectively. In the liver, 173 phosphosites were differentially regulated between wild-type and knockout mice, 20 of which were phosphorylated on multiple sites (Figure 1B, Supplemental Figure S1B, Supplemental Table S2).

**Figure 1:**
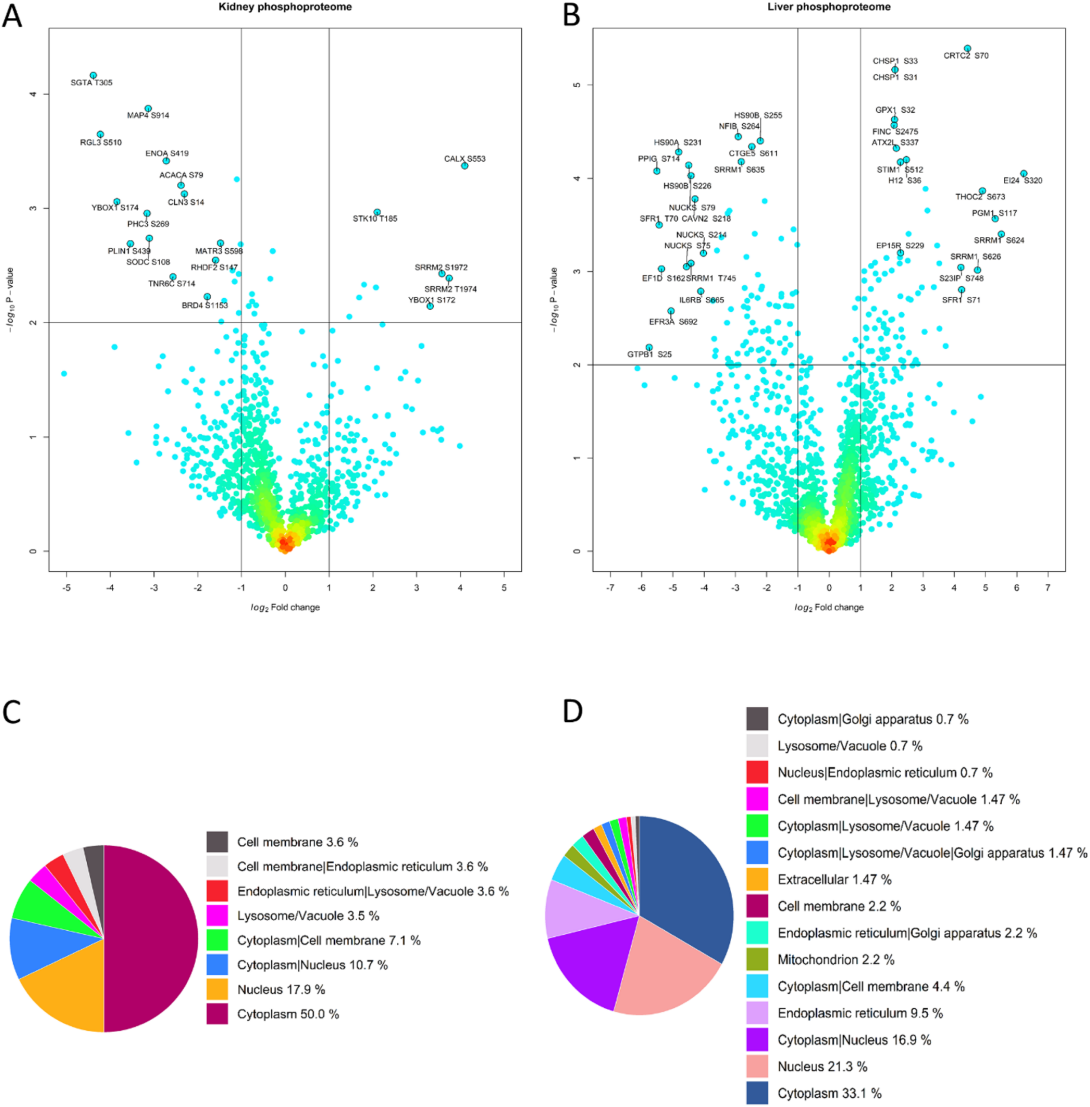
Volcano plot showing differentially represented phosphosites in legumain-null C57/BL6 mouse kidneys (A) and liver (B). The most significantly disregulated phosphosites are labeled with gene names and modified amino acid residues. Horizontal lines denote a 2-fold change in abundance, and the vertical lines denote a p-value of 0.01. Pie chart showing predicted subcellular locations of identified phosphorylated proteins as identified by the DeepLoc 2.0 algorithm in the kidneys (C) and liver (D).

To determine whether any proteins that are statistically significantly different in the whole proteome, which is not enriched for phosphopeptides, are also dysregulated in the phosphoproteome, we compared our phospho-enriched data sets with control experiments (nonenriched proteome). None of the differentially regulated proteins from the whole proteome corresponded to those that were differentially phosphorylated in the mass spectrometry analysis (Supplemental Figure S2). This confirms that the changes we observed are exclusively due to alterations in phosphorylation based on mass spectrometry data.

We utilized DeepLoc 2.0, a tool designed to predict the subcellular location of proteins based on homology with proteins in curated databases and by analyzing signal information.^47^ We determined the subcellular location of the dysregulated phosphoproteins. Half of the dysregulated proteins in the kidneys were located in the cytosol, and 17.9% were nuclear proteins. Additionally, 10% of kidney proteins were located in either the nucleus or the cytoplasm. Other subcellular locations were represented in smaller fractions (Figure 1C). Similarly, in the liver, the majority of proteins were localized in the cytoplasm (33.1%) and nucleus (21.3%), with an additional 16.9% found in both the cytoplasm and nucleus. Approximately a tenth of the proteins were located in the endoplasmic reticulum, with smaller fractions in other subcellular locations (Figure 1D).

MEME Suite with the Motif-X algorithm was used to predict kinase-substrate interactions in our data set. In the kidney, we identified motifs phosphorylated by MAPKs (xpSPx) **(686)**, AGC family kinases (RRxpS) **(66)**, and RXXpS **(117)**. Acidic kinases, such as casein kinase I and II (CK1 and 2) recognize motifs such as pSDxE (72) and pSxxD/E (79). Among the dysregulated phosphopeptides, the proline-rich motif xSPx was the most abundant (Figure 2A).

**Figure 2:**
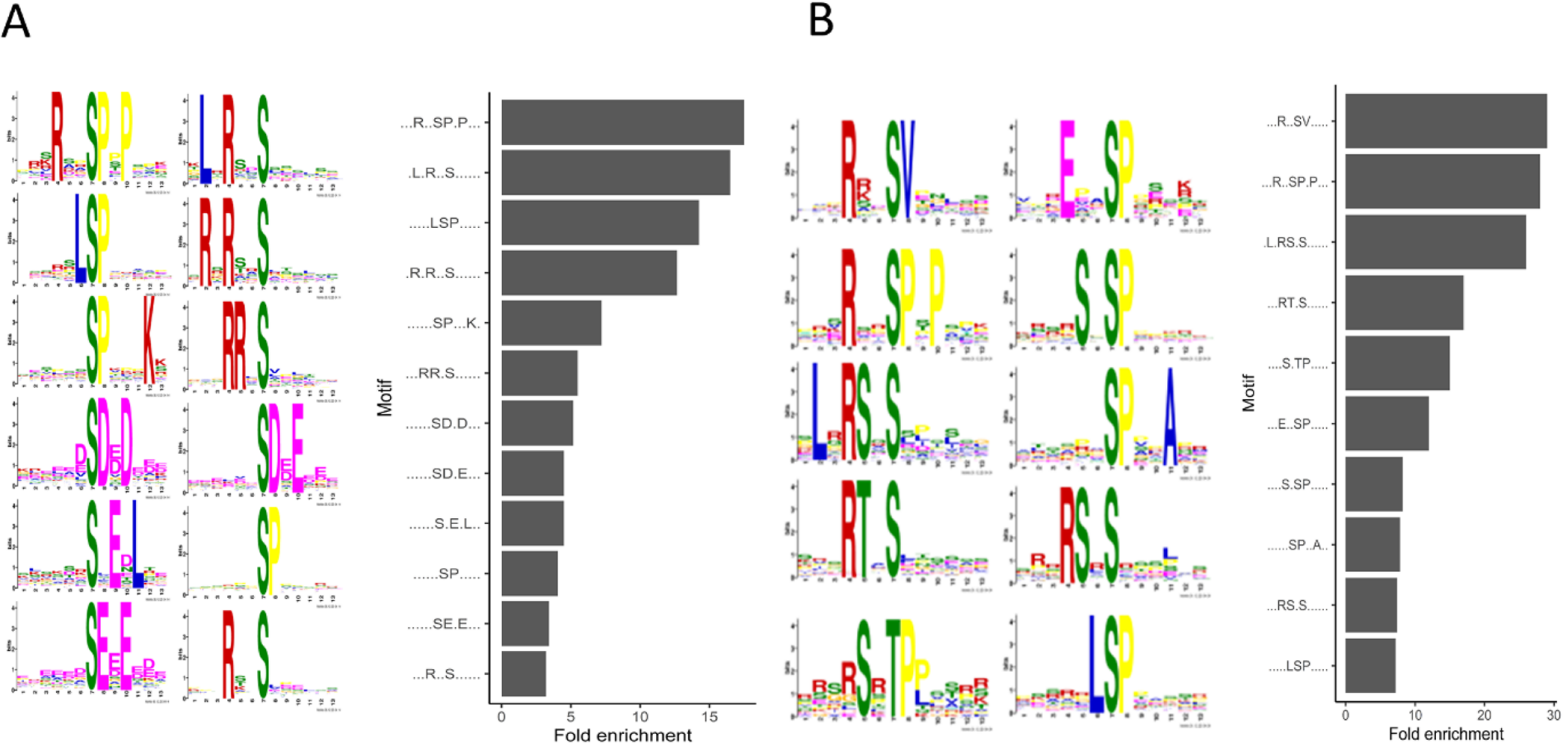
Motif analysis of identified phosphosites in C57/BL6 mouse kidneys (A) and liver (B) with their fold enrichment (total number of background peptides with central phosphorylation/number of peptides that match the motif).

In the liver, dysregulated phosphopeptides predominantly included motifs xpSPx, RxxpS, and pSxxE. In downregulated peptides, the motif xpSPx was the most enriched. Among the upregulated peptides, the motifs xpSPx and pSxxE were the most enriched. In the whole data set, the most common motifs included xpSPx (425), RxxS (361), SxxE (181), and xSx (164) (Figure 2B).

### Gene Ontology (GO) Term Analysis

Differentially phosphorylated proteins were annotated with Gene Ontology (GO) terms using G:Profiler.^48^ These proteins were classified into Biological Process (BP), Molecular Function (MF), Cellular Component (CC), and Kyoto Encyclopedia of Genes and Genomes (KEGG) pathways. In the kidney, the most represented MF terms included GTPase binding and protein binding. For BP, cellular response to stress and the malonyl-CoA biosynthetic process were the most enriched terms, while cytosol and cell junction were the most common CC localizations. Fatty acid biosynthesis was the most prominently represented KEGG pathway. Among upregulated phosphoproteins, the enriched terms included acetyl-CoA carboxylase activity, malonyl-CoA biosynthetic process, exocytic vesicle, and fatty acid biosynthesis in MF, BP, CC, and KEGG categories, respectively. For downregulated phosphoproteins, GTPase binding was enriched for MF, and dense plaque of desmosome for CC (Supplemental Figure S3). In the liver, among all differentially phosphorylated proteins, the most enriched GO terms were protein binding (MF), positive regulation of biological process (BP), cytoplasm (CC), and glucagon signaling pathway (KEGG). Among the upregulated proteins, the enriched terms included protein binding, regulation of the metabolic process, intracellular anatomical structure, and protein processing in the endoplasmic reticulum for MF, BP, CC, and KEGG, respectively. Among downregulated proteins, in the same order for GO terms, the most enriched were protein binding, catabolic process, and cytoplasm, respectively (Supplemental Figure S4).

### STRING Network Analysis

Search Tool for the Retrieval of Interacting Genes and Proteins (STRING) is a comprehensive database that includes known and predicted interactions between proteins. The interaction data are based on experimental results, *in silico* prediction methods, and data mining.^49^ We developed a complete STRING network with dysregulated phosphoproteins, five kinases identified as enzymes acting on identified phosphosites from PhosphoSitePlus^50^ (RSK, AMPKA2, ERK1, GSK3B, and CK2), and the 50 closest interacting proteins with a confidence threshold of 0.7. Functional enrichment of the largest clusters of interacting proteins was performed. The most confident GO term for the largest cluster was “RNA binding” (FDR 0.0011), while the adjacent cluster was connected with the “Proteasomal protein catabolic process” (FDR 2.97e-7). Subsequently, we identified “Negative regulation of cellular metabolism” (FDR 0.028), “Apoptotic process” (FDR 0.031), “Fatty acid metabolism” (FDR 2.87e-11), “Regulation of transcription elongation” (FDR 1.5e-4), and “Lysosome organization” (FDR 4.47e-6) in subsequent groups of proteins (Supplemental Figure S5A). For the liver, we constructed a complete STRING network from dysregulated phosphorylated proteins with a confidence level of 0.7, without additional interactors. Functional enrichment of the largest protein cluster revealed the GOMF term “Cytoskeletal protein binding” (FDR 2.65e-10) as the most enriched term. The next three clusters of interacting proteins showed GO:BP “mRNA processing” (FDR 2.65e-7), GO:BF ″cytoplasmic translation” (FDR 0.0024), and GO:BP ″carbohydrate metabolic process’ (FDR 0.0035) as the most enriched terms (Supplemental Figure S5B).

### Immunological Validation and Transcription Analysis

Unfortunately, antibodies for YBX1 phosphorylated at S172 or S174 are not commercially available. Therefore, we tested the only commercially available antiphospho antibodies for YBX1 that were specific to serine 100 (Figure 3), and we performed an immunological analysis to detect the phosphorylated site in the kidney and liver samples of mice. The findings revealed that kidney samples from legumain-knockout animals exhibited approximately five times higher phosphorylation levels, whereas no significant difference was observed in liver samples. For comparison, the difference in the whole YBX1 abundance was 2.5 times higher in knockouts. To further explore the link between legumain and YBX1, we developed a cell-based model with low basal legumain expression, where legumain was expressed in the HL-60 human cell line. In this model, a higher level of YBX1 phosphorylation was observed in the cells without legumain overexpression (control group) (Figure S6). It should be noted that overexpression of legumain slightly increased the expression of YBX1. Interleukin 10 (IL-10) is crucial for regulating inflammation, maintaining tissue homeostasis, and delaying tissue fibrosis in kidney inflammation and diseases.^51^ Knockout animals exhibited approximately two times higher levels of IL-10 in the kidneys, whereas the liver showed no significant difference (Figure 4). IL-10 is also a key downstream effector of YBX1 in kidney inflammation.^51,52^ Smaller proteins are often under-represented in mass spectrometry experiments due to lower detection efficiency, stemming from traditional proteomics workflows.^53^ This includes chemokines, and since legumain-knockout mice exhibit kidney inflammation, we tested expression levels of a few pro-inflammatory chemokines with RT-qPCR. Changes in gene expression between wild-type and knockout samples can indicate which genes are affected as a result of legumain knockout. We detected a 1.7-fold change in the expression of IL-10 receptor alpha (IL-10R) in the kidneys of knockout samples compared to those of the wild-types. IL-36a, a downstream target of NF-κB, was expressed at a higher level in knockout mice, along with pro-inflammatory cytokines CCl2, CXCL1, CCL3, and CCL5 (Figure 4). IKBα is an NF-κB inhibitor. In kidney inflammation models, elevated IKBα often reflects active or recently resolved NF-κB signaling rather than suppression. This activation can trigger transcription of both pro-inflammatory cytokines and IκBα itself as part of the negative feedback response.^54,55^ In the kidneys of knockout mice, IKBα was 2.5 times more abundant than in wild types.

**Figure 3:**
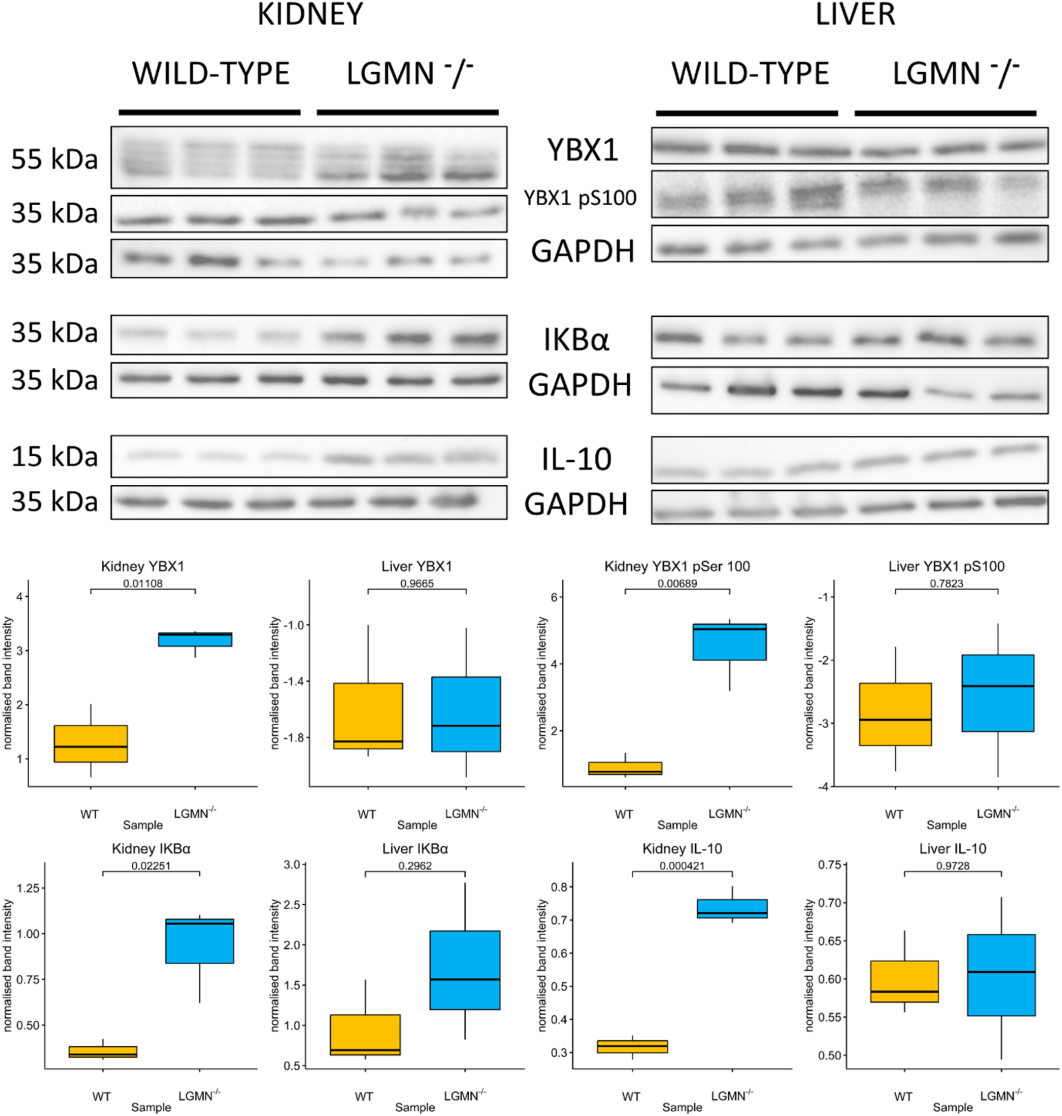
Western blot analysis of proteins YBX1, YBX1 pSer100, IKBα, IL-10, and GAPDH (as loading control) in lysates from kidneys of wild-type (*n* = 3) and *Lgmn* ^–/–^ (*n* = 3) C57/BL6 mice. Band intensities were analyzed with ImageJ software (ver. 1.49) (B), and an unpaired two-sided *t*-test was performed in R. The P-value is presented above the horizontal line above the box plots.

**Figure 4:**
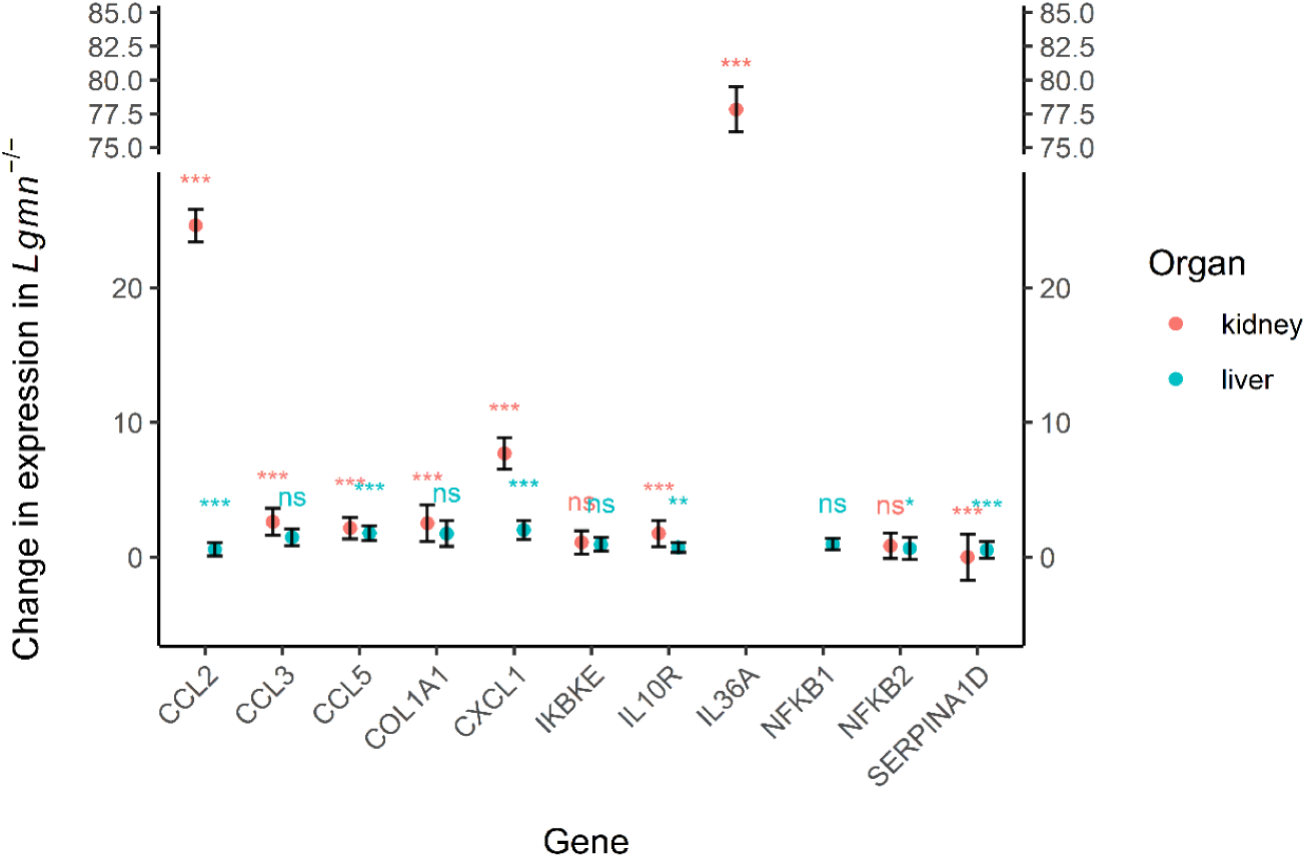
RT-qPCR expression analysis of selected genes in the kidneys and liver of *Lgmn* ^–/–^ and wild-type C57/BL6 mice. Fold change indicates an increase in expression in knockout animals. Stars indicate significance levels: ns = not significant, *. ** and *** are *p* < 0.05, 0.01, and 0.001, respectively. Dots represent the mean and bars represent the standard deviation. Expression data from the kidney is marked in pink, and from the liver in blue.

### Immunofluorescent Microscopy

Since we detected increased expression of macrophage-attracting chemokines in the kidneys of legumain knockout mice, we used immunofluorescent microscopy to compare the abundance of macrophages present in the kidneys of the knockout and wild-type mice by staining the macrophages. In the kidneys, the signals from stained macrophages (Cy3 signal) were 2 times stronger in knockout organs compared to wild-type samples, indicating a greater abundance of macrophages in kidneys lacking legumain (Figure 5).

**Figure 5:**
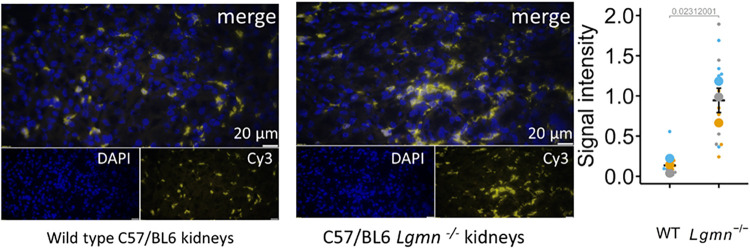
Immunofluorescence staining of C57/BL6 wild-type (left) and *Lgmn^–/–^* (right) mouse kidneys. Cell nuclei are stained with DAPI (blue), and the macrophage marker F4–80 is stained with Cy3 (yellow). Quantification was performed with ImageJ software; five areas were chosen from each sample, where the percentage of the stained area was quantified after adjustment for the color threshold and normalization of the signal to the DAPI signal. The absence of legumain results in a higher presence of macrophages in the kidneys of knockout mice. Large dots represent mean intensity values of each biological replicate (replicates denoted with colors), and small dots represent each measurement from individual biological replicates. The P-value is indicated at the top of the plot.

## Discussion

Phosphorylation is a vital post-translational modification that plays a key role in regulating cellular signal transduction. Depending on the phosphorylation site, the target protein can be activated, inhibited, or enabled to interact with its binding partners.^56^ Since the epidermal growth factor receptor (EGFR) was upregulated in legumain-knockout mice, we investigated the impact of legumain deficiency on protein phosphorylation in the kidneys and liver of legumain-deficient mice.

Legumain-deficient mice exhibit various phenotypic abnormalities affecting multiple organ systems. In the immune system, their T-cell response is slower compared to wild-type mice. In the kidneys, structural defects develop in the proximal tubules due to impaired lysosomal processing, resulting in progressive kidney damage, including reduced glomerular filtration rates and proteinuria. Furthermore, these mice exhibit symptoms typical of hemophagocytic syndrome, including enlarged histiocytes containing red blood cells and an enlarged spleen.^41^

Chronic liver disorders, including cirrhosis and fibrosis, are often associated with excessive deposition of the extracellular matrix. Legumain has been associated with fibrogenesis through the activation of transforming growth factor-β1 (TGF-β1), a major regulator of fibrosis. In chronic pancreatitis, legumain facilitates the activation of pancreatic stellate cells, resulting in enhanced production of extracellular matrix proteins.^57^

In our study, we discovered 31 differentially regulated phosphosites in the murine kidney and 173 in the liver (Figure 1). This finding is contradictory to existing pathological studies, which reported greater abnormalities in the kidneys than in the liver.^6^ In the liver, the phosphoproteomic data highlighted “GTPase binding”, “protein binding”, and “binding” as the most enriched molecular function terms. GTPases, a diverse superfamily of proteins, catalyze GTP hydrolysis to GDP (Supplemental Figures S3 and S4). Their primary role is transducing signals from membrane receptors to cellular effectors,^58^ indicating that legumain affects cellular signaling networks. In the kidneys, some of the enriched gene ontology terms were also associated with fatty acid biosynthesis (Figure S3). Fatty acids can also function as intracellular signal transducers or hormones.^59^ Proteins involved in fatty acid synthesis, such as Acacb and Acaca, were more abundant in the knockout specimens. GO further indicates that dysregulated proteins were identified as binding partners for the cytoskeleton, biotin, transcription factors, deoxyribonucleotide, and mRNA. Along with enriched terms of individual clusters of interacting proteins revealed through STRING network analysis, including mRNA processing, cytoplasmic translation, and RNA binding, our findings indicate that the observed phenotype of legumain-deficient mice may result from changes in cellular signaling and regulation of gene expression and/or translation.

Identifying enriched phosphorylation motifs can help identify upstream kinases and their pathways. Among the dysregulated phosphorylation sites, the proline-rich motif xSPx was the most prevalent in the kidneys and the second most prevalent in the liver. This consensus motif is typical for various kinases, such as cyclin-dependent kinases (CDKs), MAPKs, and glycogen synthase kinase 3 (GSK3). The RxxS motif enriched in dysregulated phosphopeptides in the liver, is strongly associated with AGC kinases.^60^ Of the 14 ACG kinase families, 8 are downstream effectors of growth factor signaling, while the others participate in diverse signaling pathways.^61^ The most abundant motif in the kidney and liver was the proline-rich SP, suggesting that legumain may affect signaling related to kinases targeting this motif. According to the Phosphosite Plus database,^50^ the most common predicted kinases based on enriched motifs in dysregulated phosphoproteins are casein kinase II, protein kinase C, and protein kinase A. Previous work in our lab^4^ identified casein kinase II subunit beta, MAPK1, and PKC as legumain substrates, highlighting legumains’ involvement in shaping the phosphoproteome landscape.

Casein kinase II is a constitutively active Ser/Thr protein kinase involved in various signaling pathways, particularly those related to PI3K/Akt and NF-κB. This kinase phosphorylates IκBα, an NF-κB inhibitor, enabling activation of the latter, as evident in our results.^62^ Furthermore, mice lacking legumain in regulatory T-cells exhibit elevated TRAF6 levels, which activate kinase TAK1, resulting in phosphorylation and degradation of IκBα and activation of NFκB.^63^ Protein kinase C, another Ser/Thr protein kinase, is a part of antigen receptor signal transduction in T-cells, B-cells, and mast cells. As legumain is involved in antigen processing, changes in phosphorylation of PKC targets are plausible in its absence.^64^ The family of protein kinases A consists of cAMP-dependent kinases that target Ser and Thr.^65^ They have a regulatory role in the kidneys, activating aquaporin 2 and water reabsorption^66^ and are involved in immune regulation, primarily as modulators of immune cell signaling and response.^67–69^

Protein kinases are crucial for regulating protein activity, which in turn affects the organism’s function. In legumain-deficient mice, some altered physiological conditions are linked to kinase activity. For instance, protein kinase A is involved in regulating kidney functionality, phosphorylating aquaporin-2 water channels, and facilitating their translocation to the apical membrane of collecting duct cells. This process increases water permeability and reabsorption, enabling the kidney to concentrate urine effectively.^66^ Receptor tyrosine kinases are involved in signaling during renal fibrosis.^70^ Several kinases, such as CDK4/6, PKC, and AKT, are involved in lysosome formation and function.^71^ In our data set, we identified several kinases through enriched phosphorylation motifs that are involved in antigen presentation, including protein kinase A (PKA),^72^ protein kinase C (PKC),^73^ calmodulin kinase II,^74^ and MAP kinase.^75^ However, information on legumain’s direct interactions with kinases is scarce. Although it is known to cleave serine-arginine protein kinase 2 (SRPK2)^76^ and participate in the initiation of the AKT pathway,^76^ there is a lack of other evidence in the existing literature.

Among the identified phosphorylated proteins, two exhibited differential phosphorylation at two sites (SRRM and YBX1). The proteins SRRM1 and SRRM2 (SRRM-serine and arginine repetitive matrix) are heavily phosphorylated, large proteins (molecular weights: 160 kDa and 300 kDa, respectively), which are located in the nuclear speck, where they play a role in regulating mRNA splicing via the spliceosome.^77^ Conversely, YBX1 is a 36 kDa protein that was found to be differentially phosphorylated exclusively in murine kidneys.

Two phosphorylated sites of YBX1, serine 172 and 174, were identified using mass spectrometry, and one additional site was detected with immunoblotting (serine 100). YBX1 is a protein that binds to both DNA and RNA, playing multiple roles, including regulation of translation and transcription, RNA stabilization, and mRNA splicing. Its function can vary, acting as either a repressor or enhancer, depending on the overall cell environment.^78^ YBX1 has 32 known phosphorylation sites located in the N-terminal alanine/proline rich domain. This domain spans approximately the first 77 amino acids and is notable for its high alanine and proline content. It is intrinsically disordered and exhibits significant variability among different cold shock proteins.^79^ The N-terminal domain is involved in cell cycle regulation,^80^ transcriptional activation^79^ and protein–protein interactions.^81^ The six subsequent phosphorylation sites are located within the cold shock domain, which is the most structurally characterized and functionally critical region of YBX1. This 80-amino acid domain adopts a classical oligonucleotide/oligosaccharide-binding fold structure with a closed β-barrel composed of five antiparallel β-strands. The CSD displays a strong preference for single-stranded RNA over double-stranded RNA or DNA, specifically recognizing the CAUC consensus sequence.^82^ The remaining phosphorylation sites are located on the largest C-terminal domain. This domain is intrinsically disordered and contains alternating regions of basic and acidic residues, termed “charged zipper” or “B/A repeats”.^83^ It is involved in DNA and RNA binding^84^ and protein–protein interactions.^79^ Only 13 of all sites were confirmed with low throughput studies, the rest only with mass spectrometry.^26,81,86–93^ Phosphorylated serine 174, reportedly targeted by casein kinase I,^85^ was more abundant in knockouts, while serine 172 phosphorylation was more abundant in wild-type specimens. Presently, there are no published studies investigating increased phosphorylation of only one of these sites, therefore it is not possible to draw direct conclusion from observed data. Phosphorylation of YBX1 on serine 172 and 174 is also facilitated by polo like kinase-1 and p90 ribosomal-S6 Kinase (RSK),^85^ regulating YBX1 translocation into nucleus in glioblastoma multiforme tumor, preventing DNA damage and apoptosis of cancerous cells.^94^ In glioblastoma, YBX1 promotes cancer cell proliferation and survival by promoting expression of several stemness genes (CD133, nestin and SOX2).^94^ Likewise, apurinic/apyrimidinic endodeoxyribonuclease 1 phosphorylates YBX 1 on both these sites in ovarian cancer, causing formation of stress granules and increasing cell survival.^95^ YBX1 phosphorylation on serine 174 is essential for YBX1 translocation to the nucleus and NF-κB activation.^96^ This finding is consistent with previous reports describing renal inflammation in legumain-deficient mice,^6^ showing slightly increased expression of NF-κB genes in kidneys, along with elevated protein abundance of NF-κB inhibitor, IκBa. The latter may be a compensatory response due to the overactivation of NF-κB pathway.^97^ To further support the activation of NF-κB signaling, RT-qPCR revealed elevated expression levels of IL-36a in the kidneys of legumain knockout mice, whose activation triggers the activation of NF-κB.^98,99^ This in turn triggers the transcription of several downstream genes chemokines (C-Cmotif) ligand 2, 3, and 5 and chemokine (C-X-C motif) ligand 1 (CCL2, CCL3, CCL5 and CXCL1).^100,101^ These inflammatory chemokines may contribute to the overall heightened inflammation state of legumain knockout mice, especially in the kidney.^102^ Interestingly, the whole YBX1 appears to migrate differently to phosphorylated YBX1 on Western blot (approximately 55 and 35 kDa respectively). This agrees with other publications using these antibodies.^103^ The disparity in apparent molecular size may hint toward possible proteolytical processing of YBX1. While YBX1 was shown to be proteolytically cleaved by the proteasome,^104^ its cleavage by other proteases was not reported either in the literature or the MEROPS^105^ database. To investigate this possibility, we searched for novel YBX1 N-termini in tissue lysates of WT and legumain knockout mice using the COFRADIC methodology^106^ (data not shown).^107^ However, we were unable to confirm any proteolytic processing events for YBX1. Although most of the existing research on YBX1 pS100 was conducted on human samples (pS102), the region is highly conserved across different species, representing functionally equivalent phosphorylation sites that regulate protein activity through the same molecular mechanism.^108^ Previous research revealed that YBX1 S100 phosphorylation triggers the secretion of several pro-inflammatory chemokines, such as CCL2, CCL5, and CXCL1. These cytokines are also released by NF-κB activation.^109^ Therefore, these cytokines could be driving factors responsible for the renal inflammation observed in legumain-deficient mice. Moreover, these chemokines attract immune cells, such as macrophages.^110^ Our study verified that macrophages are more prevalent in the kidneys of legumain-deficient mice compared with wild-type mice (Figure 5).

Wang et al.^52^ demonstrated that S100-phosphorylated YBX1 enhances IL-10 expression in inflamed kidneys, aiding in the reduction and resolution of chronic inflammatory damage. We found elevated levels of IL-10 in the kidneys (Figure 3). However, expression of the IL-10 gene in the kidneys was not detected in RT-qPCR. Conversely, expression of the IL-10 receptor was increased in legumain-deficient kidneys. Increased levels of IL-10 were also determined in the serum of T-cell- and regulatory T-cell-specific legumain knockout C57BL/6 mice. Here, IL-10 was reported to be involved in the reduction of hypertension.^63^

Increased expression of chemokines can attract the migration of immune cells, including macrophages, to the kidneys. Increased levels of IL-10 could, therefore, be an attempt to attenuate inflammation damage in the kidney.^51,111,112^

## Conclusions

Our results indicate that the absence of legumain in mouse kidneys promotes inflammation through altered YBX1 activity, which, in turn, activates the NF-κB pathway and leads to the expression of certain downstream proteins. Chemokines induce the movement of immune cells toward the kidneys, where they attempt to reduce inflammation by releasing anti-inflammatory cytokines. Our research provides information on how legumain deficiency affects the phenotype of knockout mice and opens the way toward further determination of its physiological function.

## Supplementary Material









## Data Availability

Raw proteomics data generated during this work were deposited to the ProteomeXchange Consortium with PRIDE (available at https://proteomecentral.proteomexchange.org/) and are available under the identifier PXD057650. Significantly regulated phosphosites and genes included in the study are available in Supplemental Tables 2 and 3 respectively.

## Notes

This article contains supplemental data: Supplemental Figure S1: Heatmaps of differentially phosphorylated sites identified in wild-type and knockout murine samples from kidneys and liver; Supplemental Figure S2: Comparison of differences (fold change) of whole proteins (unenriched sample) vs phosphosites in kidneys and liver; Supplemental Figure S3: Graphical representation of Gene Ontology terms enriched in differentially phosphorylated proteins identified in wild-type and knockout mouse kidney samples; Supplemental Figure S4: Graphical representation of Gene Ontology terms enriched in differentially phosphorylated proteins identified in wild-type and knockout mouse liver samples; Supplemental Figure S5: STRING network of differentially phosphorylated proteins identified inwild-type and knockout mouse kidney and liver samples; and Supplemental Figure S6: Western blot analysis of YBX1 phosphorylated on serine 100 from Human leukemia 60 cell samples, comparing the control cell line with the cell line with legumain overexpression (PDF)
Supplemental Table S1: A list of primers used in the RT-qPCR experiment (XLSX)
Supplemental Table S2: A list of differentially phosphorylated sites identified in wild-type and knockout murine samples from kidneys and liver (XLSX)
Supplemental Table S3: List of statistically significantly dysregulated phosphosites and proteins identified in this experiment, MaxQuant output tables (XLSX)

## Abbreviations

AEP: Asparagine endopeptidase
AGC: Automatic gain control
ATCC: American Type *Culture* Collection
BP: Biological process
CC: Cellular component
CHAPS: 3-((3-cholamidopropyl)­dimethylammonio)-1-propanesulfonate
COFRADIC: Combined fractional diagonal chromatography
Cys: Cysteine
D: Aspartic acid
DAPI: 4′,6-diamidino-2-phenylindole
E: Glutamic acid
ECL: Enhanced chemiluminescence
EGFR: Epidermal growth factor receptor
FDR: False discovery rate
GO: Gene ontology
HCD: Higher-energy collisional dissociation
His: Histidine
HRP: Horseradish peroxidase
IMAC: Immobilized metal affinity chromatography
KEGG: Kyoto encyclopedia of genes and genomes
Lys: Lysine
MAPK: Mitogen-activated protein kinases
MF: Molecular function
MHC: Major histocompatibility complex
MOAC: Metal oxide affinity chromatography
pS: Phosphoserine
PTFE: Polytetrafluoroethylene
PTM: Post-translational modification
R: Arginine,
RT-qPCR: Quantitative reverse transcription polymerase chain reaction
S: Serine
STAT3: Signal transducer and activator of transcription 3
TBST: Tri*sec*-Buffered Saline
TCEP: tris­(2-carboxyethyl)­phosphine
TFA: Trifluoroacetic acid
TiO_2_: Titanium dioxide
VARS: Veterinary Administration of the Republic of Slovenia
WT: Wild-type
X: Any amino acid
