# Supplementary material for: Phosphorylation of YBX1 in the Kidneys is Altered in Legumain Knockout-Mice
Source: J Proteome Res. 2026 May 6;25(6):3078–91. doi: 10.1021/acs.jproteome.6c00105 (PMC13247964; doi:10.1021/acs.jproteome.6c00105)
Supplement: Supplementary file 1 [file pr6c00105_si_001.pdf]

Phosphorylation of YBX1 in the kidneys is altered in legumain knockout-mice

Tilen Seve[1,2], Tea Sinožić[1,3], Matej Kolarič[1,2], Boris Turk[1,4], Marko Fonović[1]*

*Email: marko.fonovic@ijs.si, Phone: 00-386-01-477-3474

1 Jožef Stefan Institute, Department of Biochemistry, Molecular and Structural Biology, Jamova cesta 39, SI-1000, Ljubljana, Slovenia

2 Postgraduate School Jožef Stefan, Jamova cesta 39, SI-1000 Ljubljana,Slovenia

3 University of Ljubljana, Faculty of Medicine, Vrazov trg 2, SI-1000, Ljubljana, Slovenia

4 University of Ljubljana, Faculty of Chemistry and Chemical Technology, Večna pot 113, SI-1000, Ljubljana, Slovenia

Table of Contents:

Supplemental figure S1: Heatmaps of differentially phosphorylated sites identified in wild-type and knock out murine samples from kidneys and liver.

Supplemental figure S2: Comparison of difference (fold change) of whole proteins (unenriched sample) vs. phosphosites in kidneys and liver.

Supplemental figure S3: Graphical representation of Gene Onthology terms enriched in differentially phosphorylated proteins identified in wild-type and knock out mouse kidney samples.

Supplemental figure S4: Graphical representation of Gene Onthology terms enriched in differentially phosphorylated proteins identified in wild-type and knock out mouse liver samples.

Supplemental figure S5: STRING network of differentially phosphorylated proteins identified in wild-type and knock out mouse kidneys and liver samples.

Supplemental figure S6: Western blot analysis of YBX1 phosphorylated on serine 100 from Human leukemia 60 cell samples, comparing control cell line with cell line with legumain overexpression.

Supplemental table S1: A list of primers used in RT-qPCR experiment (XLSX).

Supplemental table S2: A list of differentially phosphorylated sites identified in wild-type and knock out murine samples from kidneys and liver (XLSX).

Supplemental Table S3: List of statistically significantly dysregulated phosphosites and proteins identified in this experiment, MaxQuant output tables (XLSX).

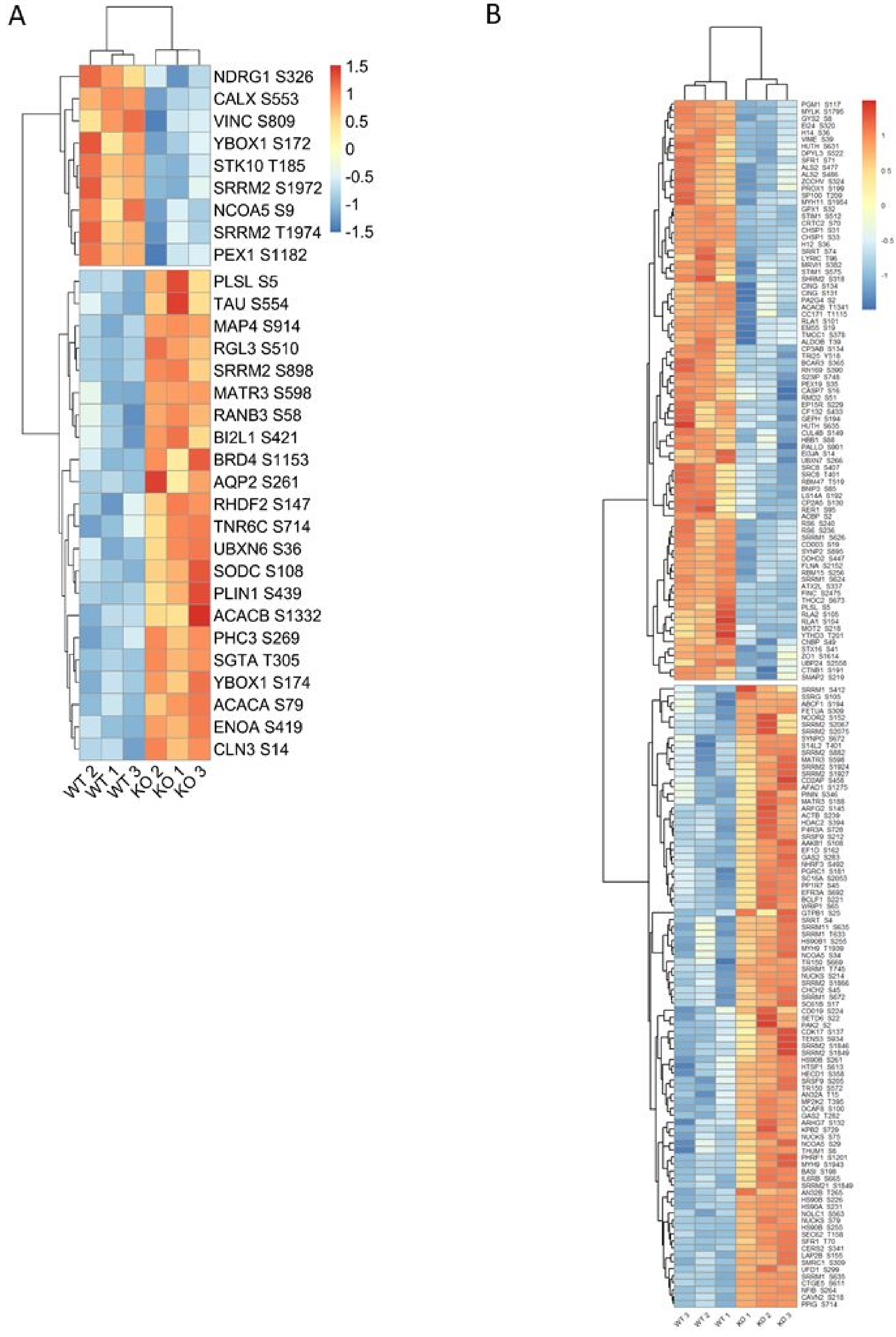
A
B
NDRG1 S326
CALX S553
VINC S809
YBOX1 S172
STK10 T185
SRRM2 S1972
NCOA5 S9
SRRM2 T1974
PEX1 S1182
PLSL S5
TAU S554
MAP4 S914
RGL3 S510
SRRM2 S898
MATR3 S598
RANB3 S58
BI2L1 S421
BRD4 S1153
AQP2 S261
RHDF2 S147
TNR6C S714
UBXN6 S36
SODC S108
PLIN1 S439
ACACB S1332
PHC3 S269
SGTA T305
YBOX1 S174
ACACA S79
ENOA S419
CLN3 S14
WT 2
WT 1
WT 3
KO 2
KO 1
KO 3
1.5
1
0.5
0
-0.5
-1
-1.5

Supplemental Figure S1: Heatmaps of differentially phosphorylated sites identified in wild-type and knock out murine samples from kidneys and liver. Heat-maps were constructed with pheatmap package in R and show under-represented phosphorylation sites in AEP -/- kidneys (A) and liver (B) with blue and over-represented ones with orange. WT 1-3 are wild-type mice and KO 1-3 are knock-out mice.

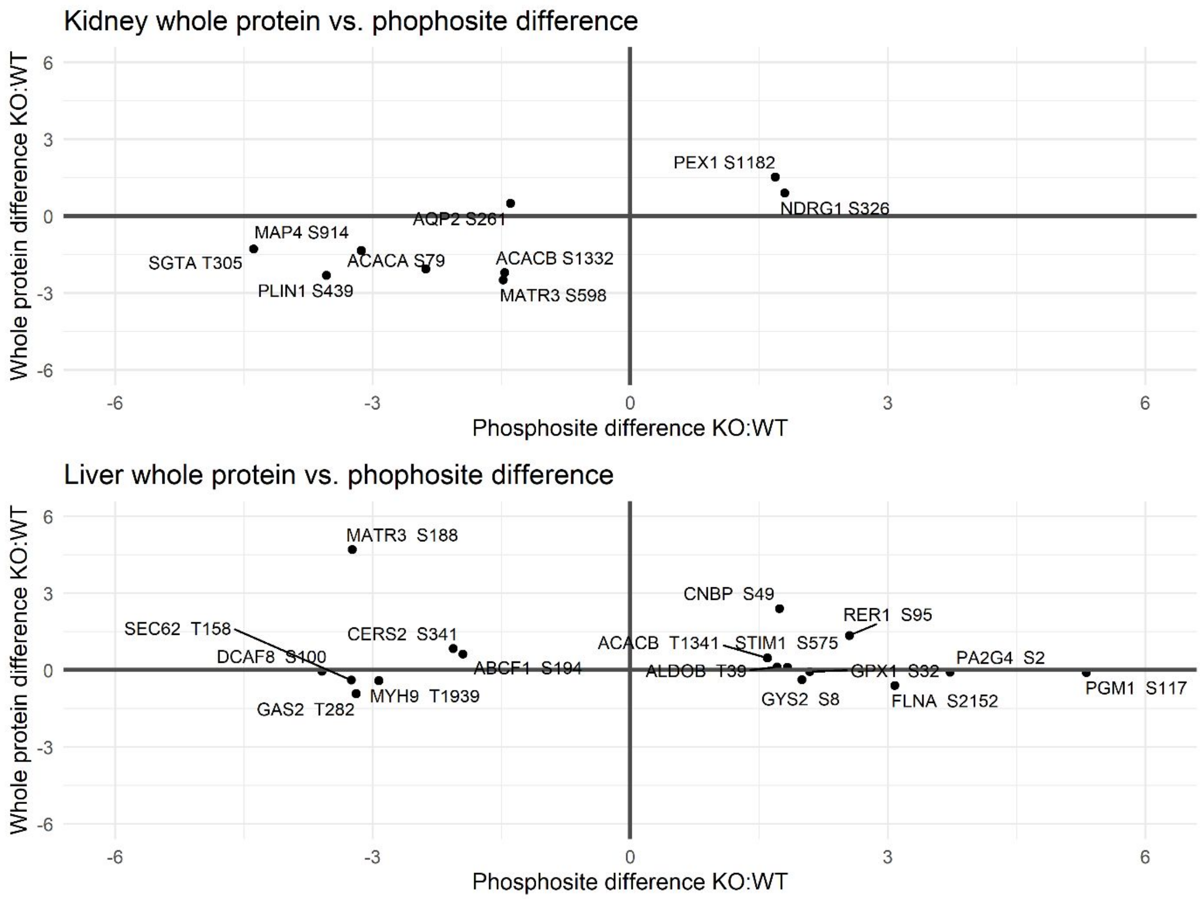

Supplemental Figure S2: Comparison of difference (fold change) of identified whole proteins (unenriched sample) vs. phosphosites in kidneys and liver. The comparison shows the log fold-change in protein signal intensities from whole proteins (non-enriched samples) and phosphosites from kidney (top panel) and liver (bottom panel). The y-axis represents the log fold change (KO:WT) of the corresponding whole proteins in unenriched samples, while the x-axis indicates the log fold change (KO:WT) of the phosphosites. Only phosphosites that were identified as statistically significantly different in the phosphoproteomics experiments and have a corresponding protein detected in the unenriched dataset are included.

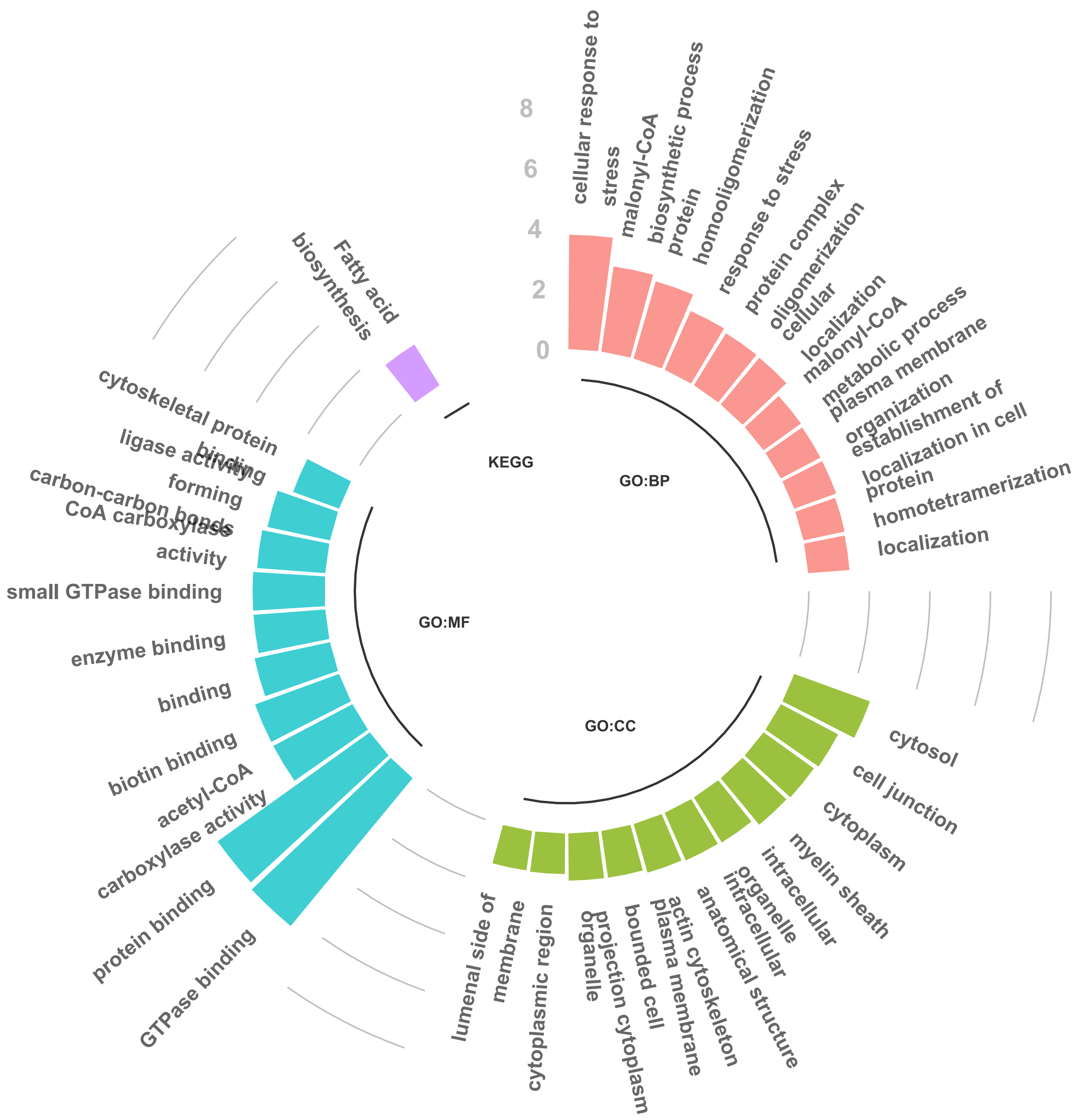

Supplemental Figure S3: Gene onthology (GO) term analysis of differently phosphorylated proteins from *Lgmn*$^{-/-}$ and wild-type C56/BL6 mice kidneys. Analysis was carried out with G:profiler. Biological process (BP), molecular function (MF), cellular component (CC) and Kyoto encyclopedia of genes and genomes (KEGG).

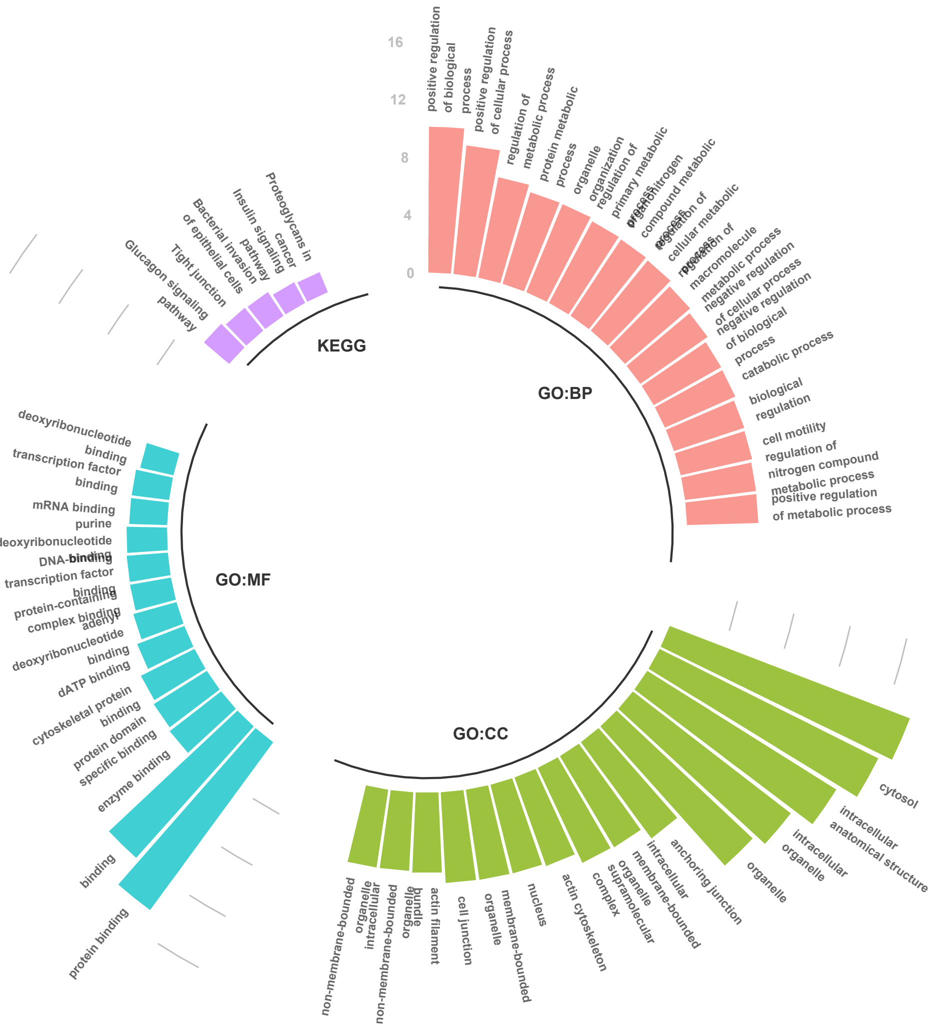

Supplemental Figure S4: Gene onthology (GO) term analysis of differentially phosphorylated proteins from *Lgmn*$^{-/-}$ and wild-type C56/BL6 mice liver. Analysis was carried out with G:profiler. Biological process (BP), molecular function (MF), cellular component (CC) and Kyoto encyclopaedia of genes and genomes (KEGG).

A

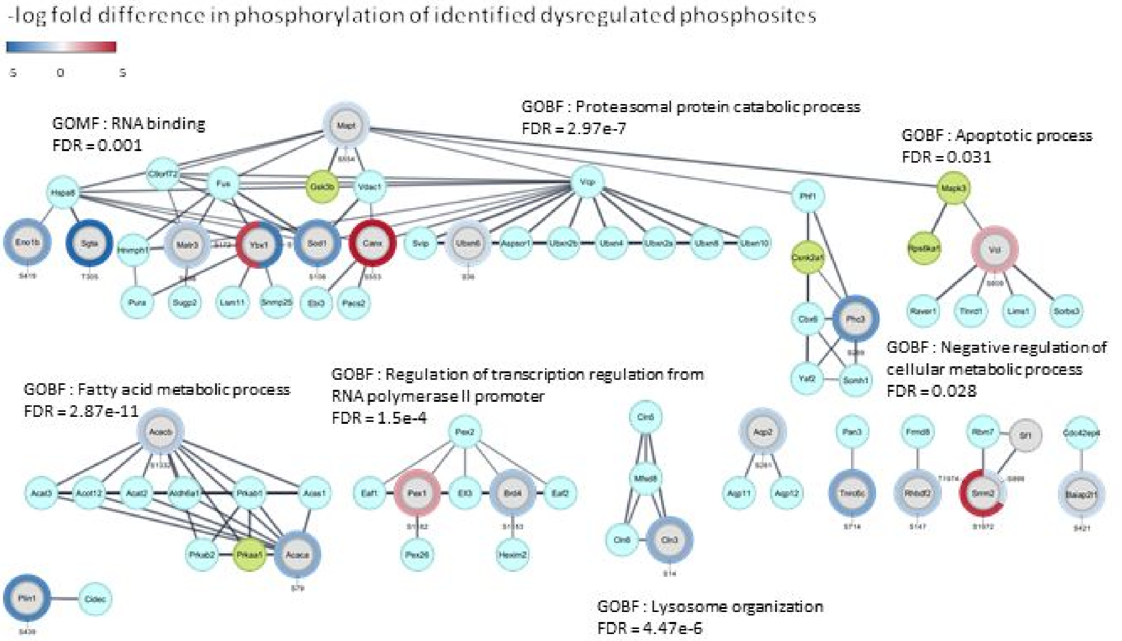
-log fold difference in phosphorylation of identified dysregulated phosphosites
-5 0 5
GOMF : RNA binding
FDR = 0.001
GOBF : Proteasomal protein catabolic process
FDR = 2.97e-7
GOBF : Apoptotic process
FDR = 0.031
GOBF : Negative regulation of cellular metabolic process
FDR = 0.028
GOBF : Fatty acid metabolic process
FDR = 2.87e-11
GOBF : Regulation of transcription regulation from RNA polymerase II promoter
FDR = 1.5e-4
GOBF : Lysosome organization
FDR = 4.47e-6

B

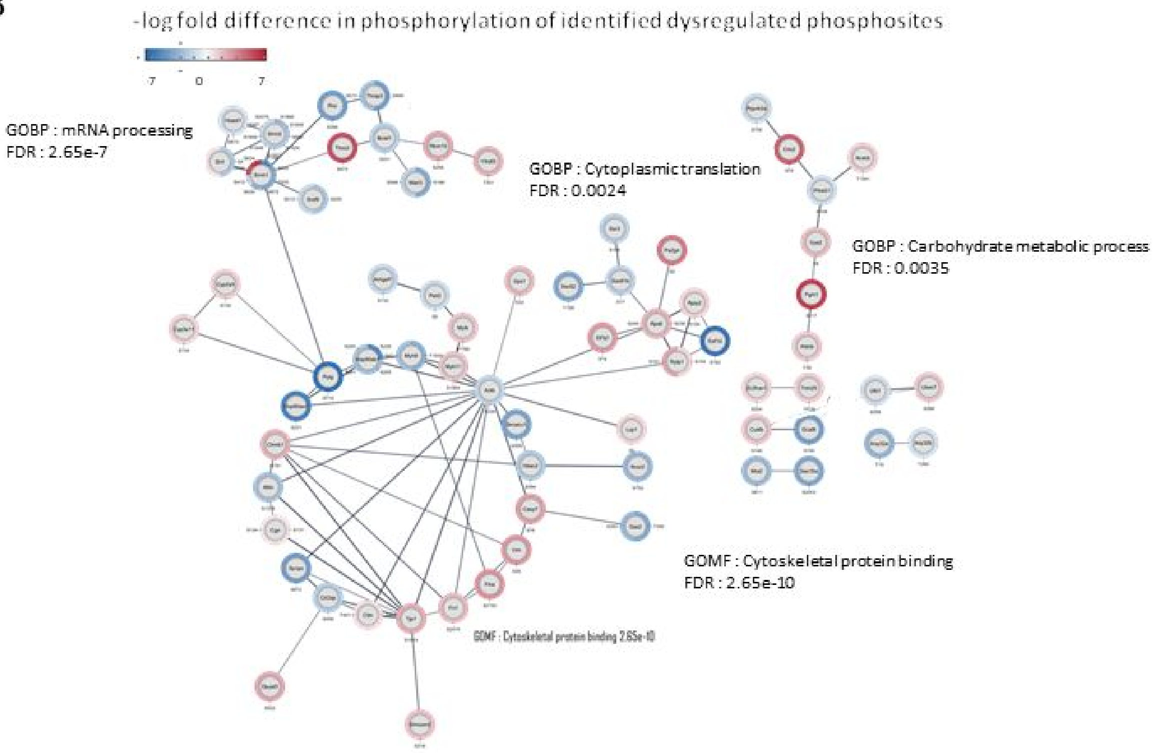
-log fold difference in phosphorylation of identified dysregulated phosphosites
-7 0 7
GOBP : mRNA processing
FDR : 2.65e-7
GOBP : Cytoplasmic translation
FDR : 0.0024
GOBP : Carbohydrate metabolic process
FDR : 0.0035
GOMF : Cytoskeletal protein binding
FDR : 2.65e-10

Supplemental Figure S5: STRING network of significantly dysregulated phosphorylated proteins in kidneys (A) and liver (B) of AEP-/- and C57/BL6 wild-type mice (grey nodes). In kidney, identified phosphoproteins are connected with their nearest 50 high confidence (confidence set at 0.7) interacting partner proteins (light blue nodes). In liver only identified phosphoproteins are shown. Phosphosites are represented with donuts enveloping the nodes, marked with phosphosite amino acid and position. Donut color presents if the phosphosite is upregulated (blue) or downregulated (red) in knock-outs.

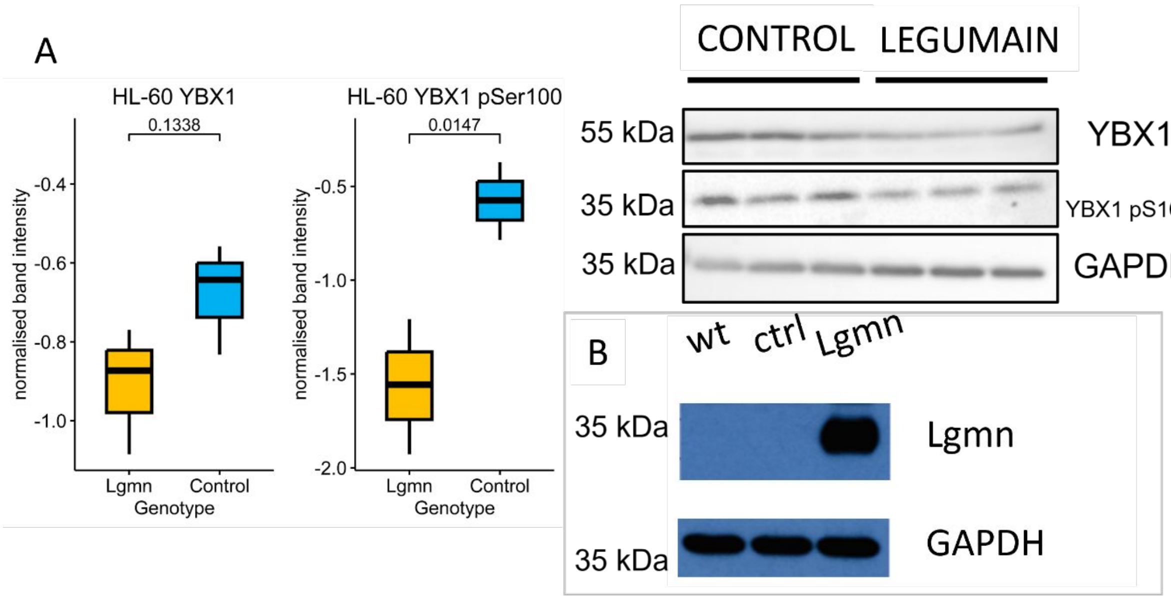

Supplemental Figure S6: (A): Western blot analysis of lysates from HL-60 cells with mock plasmid (control) (n=3) and legumain overexpression (n=3) against YBX1, YBX1 pSer 100 and GAPDH as loading control. (B): Western blot analysis of lysates from the same samples against legumain and GAPDH as loading control, showing degree of legumain overexpression. wt: wild-type HL-60 cells, ctrl: control HL-60 cells with scrambled plasmid insert, Lgmn: HL-60 cells with overexpression of legumain. Band intensities were analyzed with ImageJ software (ver. 1.49) and an unpaired two-sided t-test was performed in R, p-value is presented above horizontal line above box plots.
